# An architecture for COVID-19 analysis and detection using big data, AI, and data architectures

**DOI:** 10.1371/journal.pone.0305483

**Published:** 2024-08-01

**Authors:** Ahmed Mohammed Alghamdi, Waleed A. Al Shehri, Jameel Almalki, Najlaa Jannah, Faisal S. Alsubaei

**Affiliations:** 1 Department of Software Engineering, College of Computer Science and Engineering, University of Jeddah, Jeddah, Saudi Arabia; 2 Department of Computing, College of Engineering and Computing in Al-Lith, Umm Al-Qura University, Makkah, Saudi Arabia; 3 Department of Cybersecurity, College of Computer Science and Engineering, University of Jeddah, Jeddah, Saudi Arabia; The University of Dodoma, UNITED REPUBLIC OF TANZANIA

## Abstract

The COVID-19 epidemic is affecting individuals in many ways and continues to spread all over the world. Vaccines and traditional medical techniques are still being researched. In diagnosis and therapy, biological and digital technology is used to overcome the fear of this disease. Despite recovery in many patients, COVID-19 does not have a definite cure or a vaccine that provides permanent protection for a large number of people. Current methods focus on prevention, monitoring, and management of the spread of the disease. As a result, new technologies for combating COVID-19 are being developed. Though unreliable due to a lack of sufficient COVID-19 datasets, inconsistencies in the datasets availability, non-aggregation of the database because of conflicting data formats, incomplete information, and distortion, they are a step in the right direction. Furthermore, the privacy and confidentiality of people’s medical data are only partially ensured. As a result, this research study proposes a novel, cooperative approach that combines big data analytics with relevant Artificial Intelligence (AI) techniques and blockchain to create a system for analyzing and detecting COVID-19 instances. Based on these technologies, the reliability, affordability, and prominence of dealing with the above problems required time. The architecture of the proposed model will analyze different data sources for preliminary diagnosis, detect the affected area, and localize the abnormalities. Furthermore, the blockchain approach supports the decentralization of the central repository so that it is accessible to every stakeholder. The model proposed in this study describes the four-layered architecture. The purpose of the proposed architecture is to utilize the latest technologies to provide a reliable solution during the pandemic; the proposed architecture was sufficient to cover all the current issues, including data security. The layers are unique and individually responsible for handling steps required for data acquisition, storage, analysis, and reporting using blockchain principles in a decentralized P2P network. A systematic review of the technologies to use in the pandemic covers all possible solutions that can cover the issue best and provide a secure solution to the pandemic.

## 1 Introduction

COVID-19 is presently a worldwide [1] complex issue which affects everyone. It is difficult to simultaneously identify the disease for a substantial number of patients. Blockchain is a technique used to overcome the burden on hospitals and healthcare. Blockchain has some remarkable applications like tracking infectious disease outbreaks, donation tracking, managing crises, securing the medical supply chain, identifying fake news to allay people’s fear, and optimizing significant data analytics results. Many techniques attempt to support preliminary analysis, accurate detection of COVID-19, and abnormality localization while overcoming a lack of state-of-the-art labeled datasets. These issues still need more precise and reliable healthcare solutions, specifically in this pandemic [3]. Blockchain is based on a decentralized approach to facilitate data security and transparency between stakeholders using large-volume datasets. Blockchain ledger provides decentralization and allows every stakeholder to execute the data files all over a project [2]. This research study proposed a collaborative system based on Big Data Analytics, Artificial Intelligence (AI), and Blockchain to solve these problems.

COVID-19 affects almost everyone around the globe, but it significantly impacts children and old age. Due to the high-speed spread of this disease, people were afraid to go out freely. The major problem occurred when the physicians suggested staying in the room during the pandemic. This situation was very alarming for everyone, so the pandemic impacted the health and economy of the world. Multiple solutions are suggested by different fields, such as healthcare, pharmacy, and technologies [3]. The use of pharmaceutical and healthcare apparatus was insufficient during the pandemic, so technologies can help to overcome the burden on these fields. Artificial Intelligence is a significant field already facilitating healthcare with different approaches. Disease detection, analysis of the disease, and prevention can be performed with the help of Artificial Intelligence. Due to the large volume of datasets, the technologies that can manage the large volume of data, including storage capacity, computation, and analytics, are available to deal with big data technologies [4].

The proposed research study focuses on the current issues produced by the pandemic. These issues could be addressed easily and at low prices with the help of the latest and emerging technologies. The research problem analysis of this research study is discussed below.

### 1.1 Research problem

Due to its severe impacts on people’s lives, early detection and diagnosis of COVID-19 were necessary. The lack of resources worldwide necessitates intelligent approaches that can help identify affected people and treat them according to defined protocols [4]. Currently, available resources are insufficient to deal with the many patients simultaneously. Large-volume data analysis takes time, so big data, blockchain, and AI technologies need to be applied to accurately process the large volume of data and give solutions within defined time constraints for various patients.

### 1.2 General research gap

It is generally analyzed that modern technologies are not individually capable of confronting the complex situation of the COVID-19 pandemic. Collaborative and smart technology integration is required in this research domain to comprehend various aspects of the problem domain. The goal is to provide a secure and effective system that contributes to rapid decision-making to defy the epidemic [5]. The generalized research gap is discussed due to the need for integrated technologies like AI and blockchain to facilitate the COVID-19 solution.

### 1.3 Specific research gap

The research gap can be understood more deeply through the need for high-quality data that effectively yields accurate results using Artificial Intelligence and various big Data Analytics techniques. In addition, it is essential to know the sources of this data and use the data labeling approach to reach this end. The primary research gap was to provide the best, most reliable, and most affordable solution using the latest and emerging technologies in the field of healthcare. Especially COVID-19 was the diverse condition to provide the solution where healthcare devices and medicines could not be appropriately satisfied.

### 1.4 Research objectives

The major objectives of the research study are to facilitate early and rapid diagnosis of COVID-19 as a means of overcoming the pandemic, provide a solution to compute the large volume of data within the specific resources and use minimum resources, and provide the best-proposed architecture for the solution of AI concepts and the blockchain. This proposed architecture can be utilized to test the model.

The remaining sections of the paper are structured as follows: Section 2 presents the background based on the research problem; Section 3 discusses related work; Section 4 highlights the proposed system; Section 5 provides the discussion and conclusion for this work.

The overall research paper elaborates on the following key headings: Section 2 provides technical health background; Section 3 discusses related work, including state-of-the-art approaches; Section 4 discusses the proposed system; and the last section discusses the results and outcomes.

## 2 Technical health background

Based on the many resources, there were still issues in people’s healthcare. Intelligent, intelligent, and efficient use of resources was required to meet the global demand all over the world due to COVID-19. It is undeniable that there has been a massive increase in the data gathered and kept by firms globally in recent years. The capacity to analyze and present this information has become increasingly crucial. Big Data is a term that refers to massive amounts of data from multiple sources that saturate businesses daily. It is used to examine trends and make better decisions. With greater data storage capacity, the focus shifts from what records to preserve to how to make sense of these massive amounts of data. The Big Data industry is expected to reach 229.4 billion dollars by 2025 [6]. Despite the lack of a clear explanation, extensive data analysis has been explored in various scientific and technological disciplines, including Computer Vision, the Internet of Things (IoT), Data Analytics, Business Management, and Green Infrastructure [7].

Traditional databases, data files, email, meter-collected data, video, audio, stock ticker data, and financial transactions generated over 90% of all global data in the preceding two years. It is estimated that only 20% of this data is numeric, with the remaining 80% being non-numeric. This information may be obtained from a variety of sources and in a variety of formats. Businesses have turned to data mining and computer-aided intelligence, which have captured the public’s attention since their inception, to access this data, evaluate its usefulness, and determine how to use it to their advantage [7]. Data analysis is essential for finding, interpreting, and communicating new information and significant trends for quick, reliable, and efficient strategic thinking in various regulatory fields. This technological innovation integrates methodologies and algorithms from multiple research disciplines, including knowledge discovery, arithmetic, statistical analysis, artificial intelligence, and high-performance computation, to efficiently deal with large-scale data issues [8].

Every technology has a different level of technological richness and multi-dimensional consequences in multiple areas, including trade, government, politics, and medicine. There has been a rise of interest in big data in numerous scientific and technical disciplines in recent decades. Governments and corporate organizations are aggressively spending a significant amount of data, artificial intelligence, and blockchain technology because of their enormous potential for solving a wide range of real-world issues. The features of big data apply to data obtained from the healthcare industry, which raises the likelihood of using big data analysis techniques to improve sector services and performance. Big data analytics has many applications in the healthcare industry, such as genetics, drug development, clinical research, specialized healthcare, cancer, etc. [4]. Big data may allow for proper surveillance of the emergence of disease. Concerning adverse times of infectious disease outbreaks, COVID-19 has been unique due to the availability of digital, open, and accessible statistics with familiar figures of the breakdown of new illnesses by nation and, in certain situations, by locality. Combined with data on a person’s mobility, this dataset is excellent for combining computer modeling and AI. Computing methods allow us to view the transmission of the infection instantaneously. Big Data acquired through social networking sites and other associated unconventional data sources enables us to rebuild epidemiology stories from earlier epidemics.

Due to its widespread accessibility and acceptability, promising wearable technology will likely be a significant type of health monitoring. According to a survey performed in January 2020, 88 percent of the 4600 respondents polled expressed a desire to utilize wearable technology to assess and track their vital signs. At the same time, 47 percent of chronically sick patients and 37 percent of non-chronically ill individuals expressed a readiness to share personal health information with healthcare research groups. Fifty-nine percent of the same sample indicated they would most likely utilize AI-based companies to evaluate their health issues [9]. The use of AI in COVID-19 research has grown, particularly in the assessment, classification, detection, severity, and risk of mortality [10]. AI technology has already demonstrated its ability to track the spread of coronavirus and help high-risk individuals. In addition, it has been proven to be quite efficient in forecasting real-time infection rates by thoroughly examining preliminary data [11]. For example, citation-based analysis is a quantitative study of scholarly papers that describes trends in journals, author and journal contributions, country productivity, and details concerning research partnerships and collaborations. Bibliometric analysis may aid in monitoring trends and patterns in successful literature in various fields, including healthcare [12]. The health system may use various strategic techniques to be effective, such as sharing data and mining, machine learning, artificial intelligence, and blockchain [13]. In addition, a blockchain network is a distinct autonomous method for data collection, verification, and approval. High safety standards define it and enable patient-centered health services to be delivered, internet censorship of population safety, epidemic control, and a rapid and efficient stance procedure.

According to studies, blockchain could be used in the healthcare industry, mainly for the exchange and future management of patient data, electronic health records (EHR), and, less commonly, the distribution system management of medical equipment and drugs, the management staff of drug treatments, and the governance of drug treatments. Enhanced research, the dissemination of modern science, and the advent of new and clever medical methods have paved the way for novel techniques that have been shown to function effectively and safely. The use of tech enables the sharing of healthcare data, a crucial step toward successful integration among various EHR systems. Blockchain technology to manage EHRs can reduce clinical bias and improve healthcare outcomes. The interconnection challenge across various EHR systems might be handled using separate blockchain systems that act as cross-communication conduits [14]. AI and big data seem to have an immense opportunity for COVID-19 and other emergency preparedness situations, and its significance is expected to boost growth in the coming years [15]. Both can be utilized to instantaneously supervise the transmission of the infection, strategize and raise initiatives in the healthcare system correspondingly, spot possible specific antibodies, and increase societies’ and regions’ responses to the currently underway pandemic [16]. Such new techniques may be exploited in association with conventional monitoring in the health care system. While data analyses and interpretations are made possible, the latter reveals hidden themes and relationships, creating predictions [17].

This section has discussed the background of technical healthcare, where we can use these technologies to overcome the pandemic quickly and efficiently using a minimum of resources worldwide. The primary purpose of this section is to provide comprehensive background knowledge for the research study domain and the usage of the latest technologies, such as Artificial Intelligence, Blockchain, and others. These technologies facilitate reliable, efficient, and affordable solutions compared to healthcare services.

## 3 Related work

Various technologies have been employed to control COVID-19 disease globally, including at the technical edge [18], which uses edge technology through 5G wireless communication. Classical deep learning models address a training problem that requires a large dataset to acknowledge the findings. The proposed model used the B5G framework, which uses CT-Scan and X-ray images to detect COVID-19, and developed a mass surveillance system that monitors mask-wearing, social distancing, and human body temperature [19]. It also uses two deep learning models, ResNet50, Inception V3, and Deep Tree, to identify the presence of COVID-19 disease in CT scans and X-ray images.

### 3.1 Artificial intelligence state-of-the-art approaches

AI has been used in many studies to detect the actual disease. The proposed model in [20] focused on AI applications, including deep learning techniques to determine the COVID-19 condition. It used bioinformatic approaches to complete the whole process. Different deep learning models were used: generative adversarial network (GAN), long short-term memory (LSTM), and extreme learning machine (ELM) [21]. The AI-based platform plays an excellent role in detecting and identifying the structural behavior of the COVID-19 disease. The proposed model used various data types, such as clinical and image-based data, to get the optimum results using AI with their proposed models. Computer-aided diagnostics can early identify the presence of viruses in the human body.

The study in [22] compared various feature-based techniques with other techniques. It used some feature extraction techniques like Reset, Dense Net, Mobile Net, and NASNet to get the optimum feature set. The extracted features are fed to various classifiers to check the accuracy by classifying between normal and COVID-19 [23]. Although the system can detect COVID-19 automatically by applying multiple deep learning methods, it only detects COVID-19 disease data analytics, which can contribute significantly to identifying major affected areas. Various techniques were applied to identify and analyze COVID-19-affected areas. The proposed method in [24] used big data analytics methodology and focused on how to solve contemporary organizational issues. This technique described insights about prediction and analytics. It focused on using big data analytics to study and find solutions for the COVID-19 crisis. It helps policy-makers and managers who are performing their duties for the problems. A convolutional neural network (CNN) can effectively identify the structural behavior and the disease categories. The proposed model in [25] is based on the transfer learning approach in the deep learning field. The transfer learning approach is used to detect COVID-19 from chest X-ray images, which minimizes the need for manual COVID-19 detection. This automated deep learning model reduces reliance on the human eye, offering more straightforward, easy, and accurate COVID-19 detection.

The latest advancements, such as molecular and computational approaches, have played a vital role in identifying coronavirus. In addition, AI information and communication technologies also play their part in finding the optimum solution. Meanwhile, big data analytics detects and predicts coronavirus. The proposed model in [26] discussed big data analytics, which handles unprecedented data collected from public surveillance. This data is used to forecast trends and typical coronavirus situations. The proposed model in [27] used common techniques like data mining on big data and deep learning models. A vast dataset was used on which big data analytics techniques were used to identify coronavirus. Data mining and machine learning techniques were used for detection purposes. The transfer learning approach was used, which learns from a smaller set of samples and then is applied to the whole or larger dataset. Hasan et al. [28] proposed a technique to overcome the pandemic by tracing contacts of known COVID-19 cases. Various other methods were used to reduce the pandemic and help to overcome the disease. At the same time, accurate information was needed to trace those suffering from the condition using their contacts. There are numerous ways to find contacts, but this study proposed using decentralized blockchain techniques.

The user will first provide their information using digital devices and then use some methods to save their knowledge in the central repository. Using blockchain, the data warehouse is decentralized for all stakeholders, allowing them to find patients quickly. Rajesh et al. [29] proposed a solution based on blockchain privacy preserved federated learning model. It is recognized that the COVID-19 pandemic is almost worldwide. The dataset obtained has considerable data and needs to be more easily managed using traditional methods. The study suggests a blockchain-based solution since AI techniques still do not meet actual requirements. The data is decentralized and encrypted to avoid any loss of data. The blockchain ledger decentralizes data security and privacy. Two methods are applied: training the model by novel capsule network and classifying COVID-19, and, in the second step, using homomorphic encryption to secure the data. This is the new approach using blockchain to organize and secure the data.

### 3.2 Deep learning and IoT state-of-the-art approaches

Rajesh et al. [30] proposed a blockchain-based approach to handle the diverse situation of COVID-19. The Blockchain-Envisioned-based approach for the software services in the multi-swarming scheme represented the proposed approach. With the usage of 5G, human intervention will be reduced to a very low rate, and software usage will be reduced with the help of blockchain technologies—communication between people during the COVID-19 pandemic. The proposed approach used the 6G technology framework with smart communication devices. The proposed model was outperformed, as seen in the visuals and statistics.

Darshan et al. [31] discussed the most valuable approach to handling the COVID-19 pandemic. Multiple issues during the pandemic were faced, destroying the world economy. They were necessary to handle the worst situation and needed to provide the best solution. The proposed approach used artificial intelligence, big data analytics, and the Internet of Things, integrating all of them to provide the best solution. Bidirectional Long Short-term Memory (LSTM) was used to train the model, and these trained models were used for testing purposes in the solution. The boost scheme outperforms the proposed solution based on the results shown in the visuals and statistics.

Sudeep et al. [32] provided an AI-based solution for the COVID-19 disease detection and prevention approach. The proposed model used the ARIMA, LSTM, and MRD approaches to deal with the diverse situation in the pandemic. The proposed scheme, D-espy, outperformed and obtained more than 92% prediction accuracy values. The approach was totally based on Artificial Intelligence technologies and trained the large volume dataset. Further, these models were used for the test case.

Personal security and data protection were the significant challenges in the COVID-19 pandemic. NY et al. [33] proposed an approach to provide the best security features during a pandemic. The Korean government checked the security concerns in the personal data usage process. The proposed research study used the model to ensure that personal data was available to the healthcare apparatus during the pandemic. Safety governance [34] was the major problem during the pandemic, so the discussed approach facilitates the best solution to provide safety solutions. The proposed approach designs the best methodology to manage the whole environment. ICT and Big Data technologies linkage [35] were the revolutionary work in which the Korean case study was focused on addressing the security-related issues of personal and data-provided institutions.

### 3.3 Current state-of-the-art approaches COVID-19 examples

State-of-the-art approaches were reviewed to see the current status of the ongoing research on vaccines and traditional medical techniques for COVID-19. E Irmak et al. [36] discussed COVID-19 disease detection with the help of deep learning approaches. Convolutional neural networks were trained on the state-of-the-art dataset to understand the primary reasons. Based on that approach, the model was successfully and efficiently tuned for disease analysis, such that the evaluation measure shows its performance on the real-time dataset. Tools are primarily used to detect pandemic-type diseases like COVID-19. Udugama et al. [37] proposed the best tools for disease detection. These proposed tools help analyze the dataset efficiently and provide valuable insights into the dataset. Artificial Intelligence and the latest tools help diagnose diseases in healthcare.

DeepCov19Net was introduced by Demir et al. [38] for disease analysis and detection in diverse situations. Deep learning is the approach that helps to find the complex symptoms of the disease efficiently. Deep learning models like UNet, ResNet, LSTM, etc., outperformed on that type of disease. Best evaluation measure values prove the quality of the model performance. CNN is also the deep learning-based approach used to detect COVID-19 disease, and it has performed well in diverse situations. Rashi et al. [39] discussed the valuable approach of using the CNN-based model in the deep learning-based approach. X-ray image dataset was used to train the model, and then the trained model was tested with the unseen image dataset.

Irmak et al. [40] added a novel deep learning base model in the field of healthcare, using state-of-the-art datasets to train the models. The model’s accuracy values show the best performance in the real-time test dataset. Deep learning approaches outperformed in many places, especially in the healthcare domain.

Based on the above multiple techniques applied, it can be concluded that Artificial Intelligence outperformed with the help of deep learning models. IoT devices were used to collect a valuable and reliable dataset for the model training. So, it was decided to show the addition of new merging technologies for the security of the dataset. Blockchain with a security feature helps to protect the patient’s dataset, and deep learning is best for model training.

## 4 Proposed system

**Fig 1:**
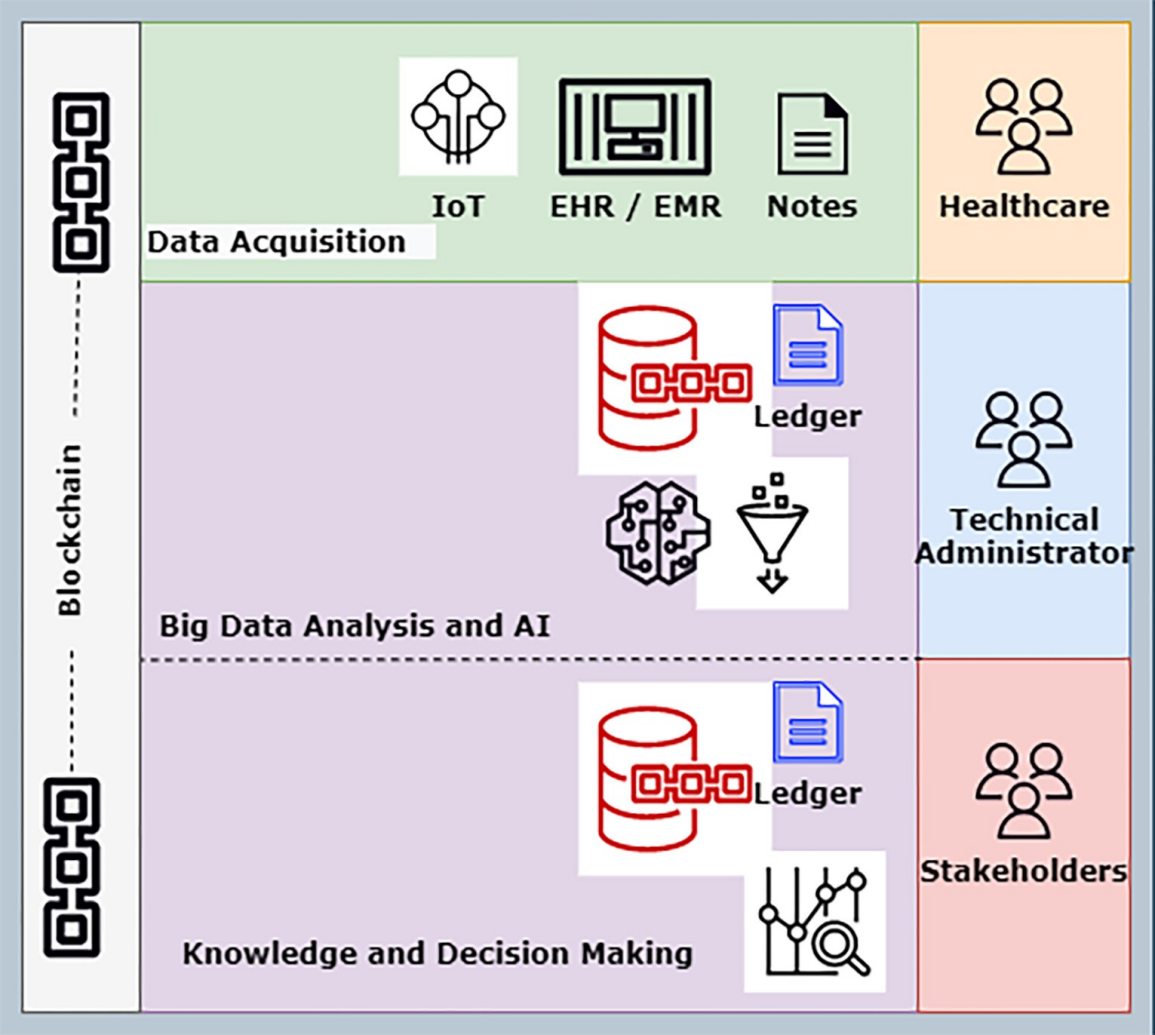
DataOps pipeline architecture for proposed model.

The inner core of the model implements Big Data and AI techniques to detect and analyze COVID-19 using CT-Scan and X-ray images [41]. The Convolutional Neural Network (CNN), DarkNet algorithms, and deep-learning techniques are used for this purpose. The implementation focuses on employing the DataOps concept to manage the whole model by applying the pipelines shown in (Fig 1). The proposed model in (Fig 1) clearly defines each step elaborated in the development.

The proposed pipelines are discussed below, which were used during the development of the system:

- Data Acquisition Pipeline
- Data Analysis Pipeline
- Innovation or Development Pipeline
- Execution and Knowledge Pipeline

The proposed system is designed to detect and analyze COVID-19 based on the DevOps concept implicitly featuring DataOps.

### i. Data acquisition pipeline

The model development pipelines start with acquiring data from different sources, as the high-level architecture illustrates, whether through the Internet of Things (IoT) devices or medical notes. Healthcare staff performs user interactions with this data-gathering process. Moreover, open-source datasets are available to train machine learning models. Kaggle^1^ is the best open-source data repository, which will be used to train, develop, and analyze the model efficiently. Other sources^2^ of COVID-19 datasets are available now with quality datasets. So, the other way to collect datasets from healthcare, hospitals, and health provider institutes is initially expensive, time-consuming, and utterly unreliable. The open-source dataset could help us access it easily without cost, even though multiple people have already researched it, proving its quality. In data acquisition, the system will propose to get an open-source dataset compared to those obtained from health providers. Both approaches are shared with the researcher to collect the data; either he can use the open-source dataset to train the models or collect the dataset using IoT devices installed in the hospitals or healthcare places.

Once the data synchronized from the medical staff or stakeholders at the hospital level is completed, and all the inputs from the IOT devices are committed to the hypervisor Ledger in the blockchain, various diagnoses of the patients suffering from COVID-19 are restored. The data acquisition completes with the block commit in the blockchain responsible for holding the data from this level.

### ii. Data analysis pipeline

In the next stage, all data collected is stored in database repositories to conduct analysis processes and artificial intelligence techniques by data staff and developers. Finally, results are visualized, which supports any decision-making or future directions and trends. It is notable from the architecture that all the data sharing between different layers will be achieved using blockchain technology, which will enhance the data security, decentralize it, and make it transparent to all those dealing with the system in an intelligent collaborative framework. Dataops used to manage the whole project in their defined pipeline. To cover the different technologies and approaches dataops pipeline was used for the easy management model.

Data security is a critical challenge; we must provide the best quality data protection approaches. To ensure the security and privacy of the data collected by the Internet of Things devices or collected by the open-source at every layer in the prescribed model, the proposed system recommended using blockchain security features. The blockchain security feature is reliable and provides the best data theft and manipulation security. In all the exclusive layers in the prescribed diagram above, the blockchain model receives the input from the layers and appends with the existing decentralized data block. This data block is responsible for holding the versions that were archived or created at the previous time. The finalized versions are connected with smart contracts between various stakeholders and are updated to the final level. The data is finalized and completely connected in the form of an operational management pipeline that’s committed and stored in the decentralized level blockchain. This ensures that the data block, available as the final version, is accessible globally into the P2P network, which handles and takes care of all the blocks. For a specific country and a geographical location, the data blocks are managed with the help of a P2P network, depending on the vast area network structure.

During the data analysis, the data noise, including inconsistencies, non-aggregation, incomplete data, and distortion of the data analysis in this section, was resolved during the model development. Data preprocessing is the technique where we resolve these issues in the dataset, such that in the data inconsistencies, the data managed to make them all in the same pattern. In contrast, data integration creates issues in the data column so that it can be aligned with all the same columns.

### iii. Innovation or development pipeline

The next level comprises the information that is analyzed with the help of the information passed by the initial data acquisition layer. The information from the previous layer gets to the analysis bundle, where various analyzing techniques are used. The compressed information is simply available in the data block. This information is compressed with the help of various data storage techniques discussed in the upcoming section. However, it must be noted that various cloud service providers give services responsible for handling and analyzing such data once the information is analyzed from the cloud service provider data model. It can be further sent as a data block to the upcoming layer. It is also worth mentioning that the information taken from this layer is committed to the block by the database administrator or technical administrator. Analyzing the data acumen related to the previous step is one of the most critical steps in this process.

### iv. Execution and knowledge pipeline

Once the data is analyzed, the model becomes responsible for making decisions with the help of various artificial intelligence techniques. These techniques are also available at various cloud service provider engines. However, for the sake of simplicity for the model, we would analyze the information with the help of traditional AI-based techniques. Once the knowledge base is ready and the dataset is verified, the final information, which is in the raw format, can be considered to be helpful for decision-making factors; the decision-making can be done with the help of various robust AI techniques and algorithms, these algorithms are specified in various levels, and many of them are very well-known. An algorithm can be chosen depending on the class of data and the dataset size. The results from applying these algorithms to the dataset yield proper decisions, which go to the stakeholders. We must mention that. Once the decision arrives, the final data block comprising the information should be updated in the blockchain across the P2P network. It is also necessary that the decentralized data makes a replica and a copy across all the nodes in the blockchain.

### 4.1 Smart collaborative model for analyzing and detecting COVID-19

The smart, collaborative model design includes four pipelines for the complete execution of the architecture, each with its importance and values. These pipelines are of immense importance in this model. The model specified in this study can help derive various features

- **Dataset:** This is a Data Acquisition Pipeline in which Doctors and Healthcare staff are stakeholders who provide the data. The data may be collected via IoT devices or manually as medical notes. These medical notes are updated by stakeholders, such as doctors and staff members responsible for taking care of the patient. However, in some instances, the physical features like blood pressure, heartbeats, rate, oxygen level, cardio study, and more medical properties of a patient can be studied with the help of specialized IOT devices. The information obtained by these devices gets synchronized automatically at the cloud service level provider.
- **Data Analysis:** This Data Analysis Pipeline conducts pre-processing steps to remove outliers and apply machine learning techniques to analyze and visualize the dataset. The patient data then passes to the next layer for further processing. Data analysts are stakeholders in this layer. Data analysis can be done with the help of various AI-based techniques, which involve machine learning and neural networks. The information received from the IOT devices can be analyzed across the Channel with the help of specific deep learning algorithms.
- **Innovations:** This is the Development Pipeline in which a model is developed to process the patient data and detect COVID-19 from data sources. All the training and testing models are developed in this phase. In the end, a classification between COVID-19 and normal is made. After the model’s training, the data from various patients and other information channels is loaded into the model. The training data model tries to learn from the existing data and the new raw data from the previous step. Artificial intelligence plays an essential role in this regard. Because the model trains itself and learns from new information, a decision can be made for the patient once the data is received and analyzed.
- **Operation:** This is the execution and knowledge pipeline used to check the accuracy of the architecture. The data architecture, completed at this stage, yields a result representing the patient’s state. The global availability of the result is one of the critical factors that help the patient and the community. During the pandemic, various research studies were conducted, both biological and technical, to identify the solution for the disease. However, data from around the globe were required to develop and design this solution. The model prescribed in this study tends to submit results at global boundaries with the help of a blockchain network. The hyper Ledger, responsible for committing all the transactions from various stakeholders in this operation, provides quality and sustainable data. This data source is very secure and proper, with a concise definition of the information.

**Fig 2:**
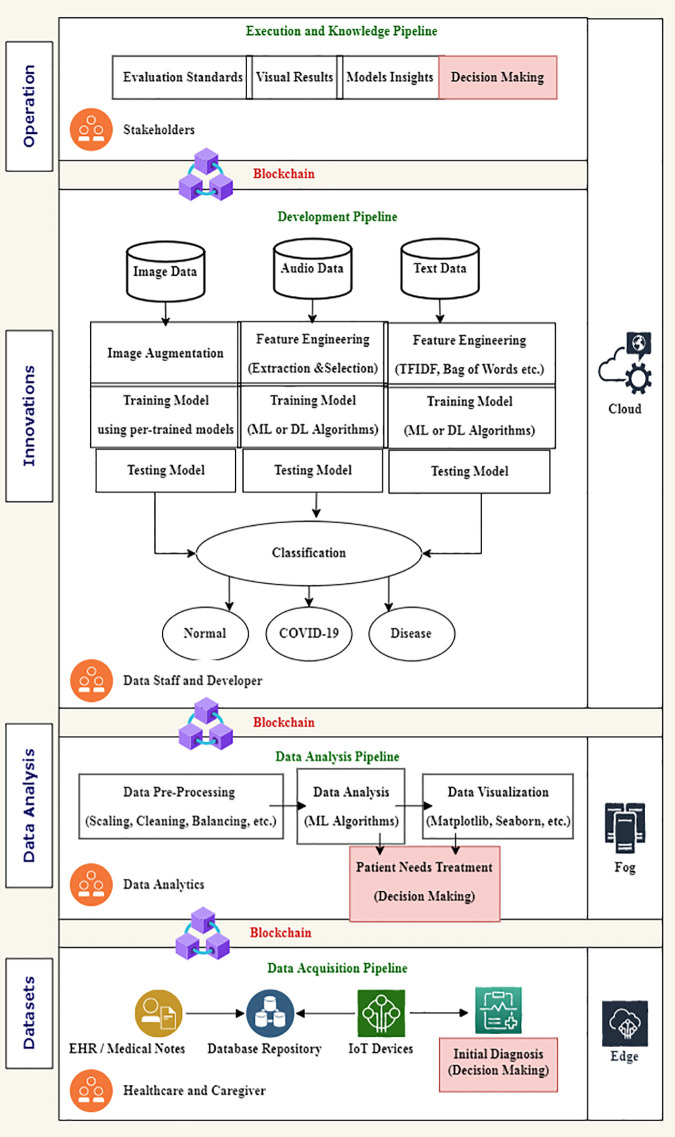
Proposed architecture.

All the pipelines mentioned above explain the complete architecture, and each phase clearly understands its role. DataOps manages the whole architecture under a single umbrella. It gives easy access to each phase and clearly defines the concept. (Fig 2) shows the smart, collaborative model for analyzing and detecting COVID-19.

### 4.2 Detailed architecture of the proposed model

The detailed proposed architecture was based on a comprehensive research study where gaps were identified in the research problem. To address these issues, the proposed model has designed the architecture that can be applied to provide the best solution in this domain. Each stage of the proposed model clearly shows its purpose. The proposed architecture is compulsory to apply the process to provide the solution.

Based on the proposed research model, the critical option was to provide privacy and confidentiality to the patient’s dataset. Patient data protection was needed to protect efficiently such that in the proposed model, blockchain security features were used to keep the dataset encrypted. Blockchain security feature provides the best services to protect patient data where only authorized users can view the records. The purpose of using blockchain in the proposed model is to manage the large dataset gathered from IoT devices or open sources. A decentralized approach is used in the blockchain to protect the data, and an encryption approach is used. These two approaches help to facilitate user data protection for any type in the healthcare domain. The Proposed model suggested using these types of approaches to facilitate the users.

Data management is the key to processing, analyzing, and computing data. Due to emerging technologies, the data volume is going to increase day by day. Big Data has specific indications that it is growing exponentially over time [42]. Big Data differs from traditional data because it has vast volume, variety, and velocity [43]. It has heterogeneous formats like structured, semi-structured, and unstructured data [44]. Advanced algorithms with machine learning and deep learning techniques are required to process or execute a large amount of data. Traditional business tools are needed to process complex and large amounts of data [45]. According to Gartner [46], Big Data is information assets of large volume, variety, and velocity that require very high computation, cost-effective, and high-performance algorithms for decision-making and checking data insights [47].

Datasets is the first and primary step of the proposed model is receiving information from various sources. As shown in (Fig 2) above, the healthcare services try to collect the information from the data acquisition pipeline. This acquisition pipeline handles the data with the help of edge computing. The edge devices are connected to the IOT-enabled services and various data repositories in different formats. Edge computing is essential in generalizing and optimizing the inputs given by various devices. The edge gateway devices convert the information from these IOT-enabled devices to a format the data pipeline understands. Once the information has been collected from this dataset. The acquisition pipeline tries to submit the information to the blockchain with the help of smart contracts. The experimental setup for the blockchain smart contract exchange is explained in the later part of the study.

**Fig 3:**
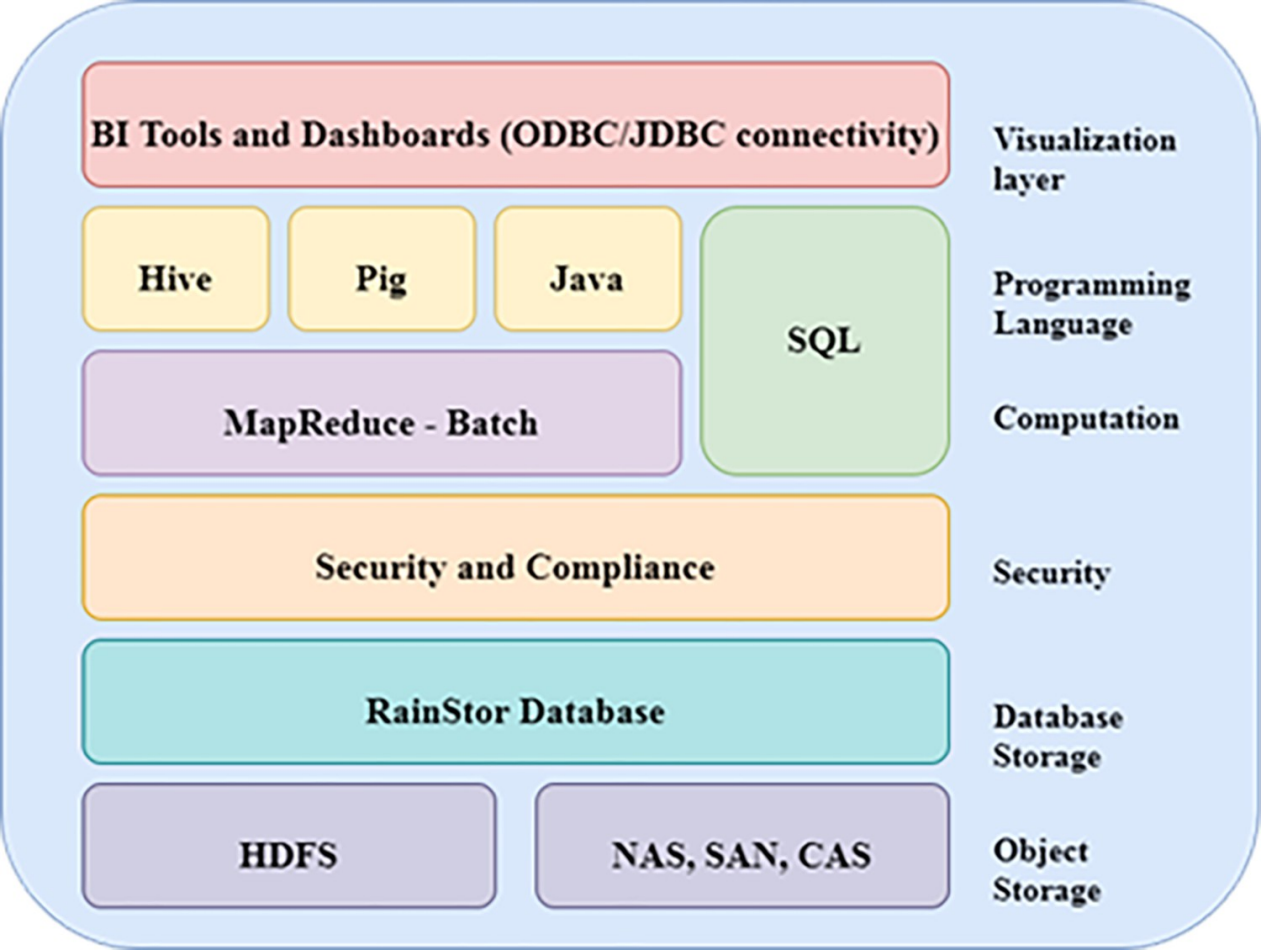
Layered architecture for the model processing components.

**Fig 4:**
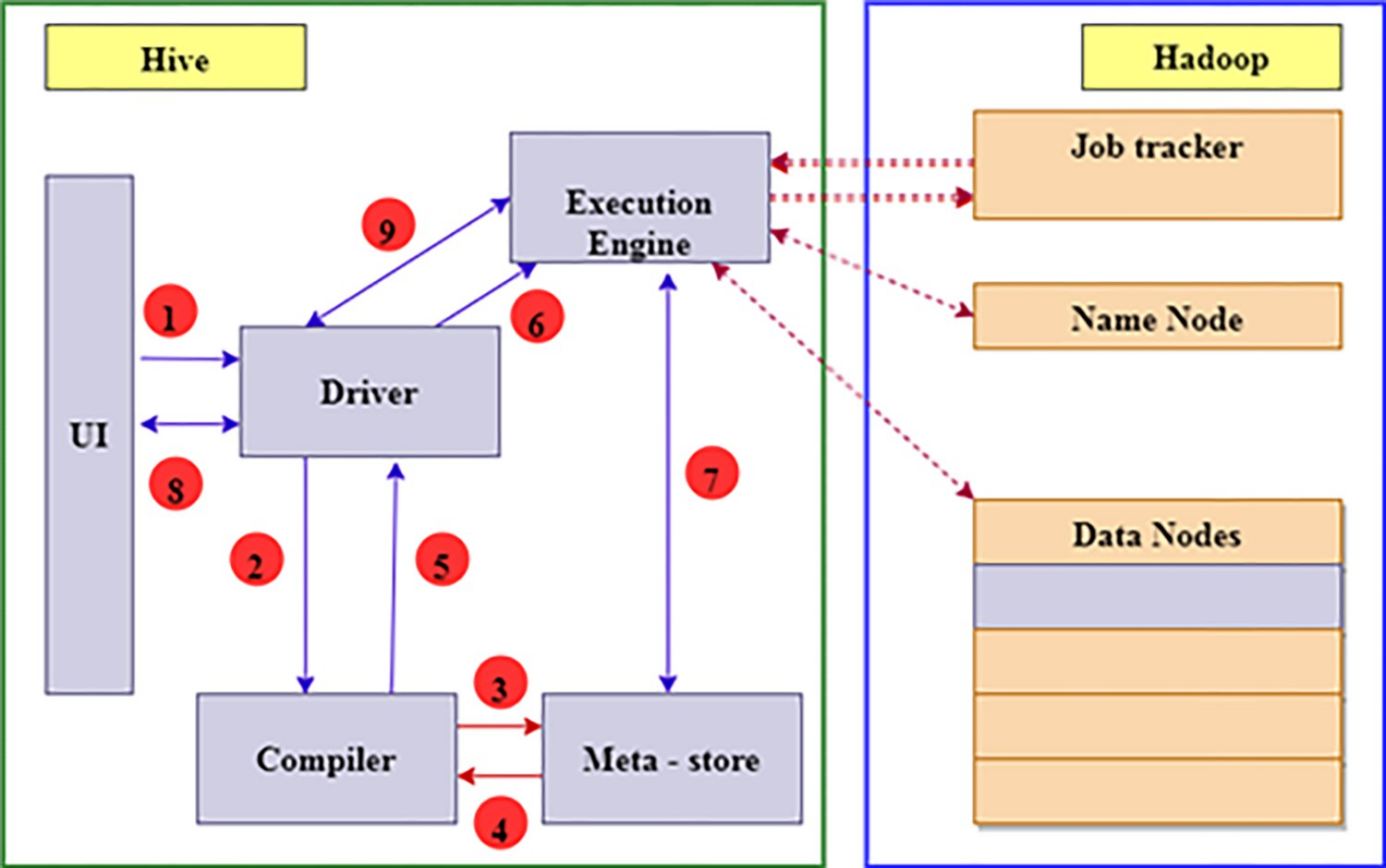
Data storage architecture for Hadoop and hive integration (A proposed model component).

The data architecture, which is followed in this study, comprises the information collected from various data sources. Various data storage technologies are available as open-source services in cloud computing technology. Table 1 below represents a strong comparison between various data storage technologies. The prescribed model can ensure that any data comparison and storage can take place with the help of well-known services like Hadoop, MongoDB, RainStor, or SplunkHunk [48]. (Fig 3) above depicts the RainStor as a database layer in the layered architecture for the model processing components. The (Fig 4) below represents the mechanism between Hadoop and Hive framework for data architecture management [49]. The information flow and various steps in the prescribed framework guarantee the system statistics and other information management. The engine for managing the driver and jobs collaborate to preserve the data in an organized way. The proposed model is open to all types of data storage frameworks. However, due to the map’s reduced capability of the Hadoop framework, many data stores rely on this [50].

**Table 1:** Technical comparison between data storage technologies.

No.	Technology Name	Key Terms
***1***	*Hadoop*	*Hadoop uses a Distributed File System and MapReduce Programming Model*
***2***	*MongoDB*	*MongoDB using MapReduce and Aggregation Framework/Architecture*
***3***	*RainStor*	*RainStor has integrated its architecture with Teradata with five layers*
***4***	*SplunkHunk*	*SplunkHunk Explores and Visualizes data using Hadoop clusters and SplunkHunk database connected via API*

There are a couple of more services not prescribed in Table 1. However, the data analysis done at the edge gateway level enables either of them to use the information in the standard data formats [51].

**Fig 5:**
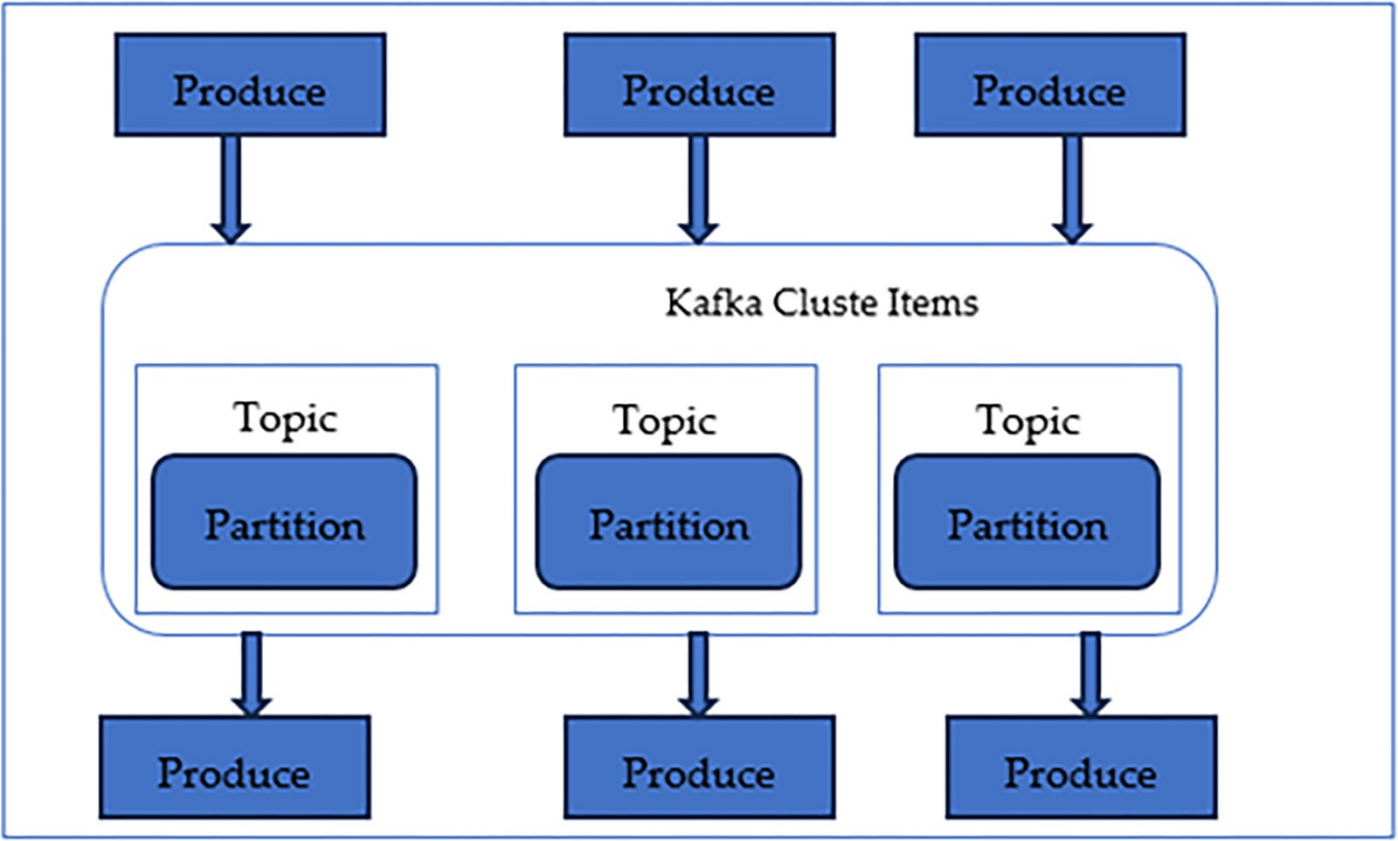
Data analytics component of proposed model making use of Kafka cluster.

Data Analytics: After collecting the data from various sources and converting the format in the prescribed analytics part, the analysis pipeline takes responsibility for further processing of the data. This data is preprocessed with various techniques like scaling, cleaning, and balancing the information in the prescribed format [52]. The next stage of the data analysis pipeline makes the information passed through the machine learning algorithms, which will be responsible for decision-making. This is one of the most critical steps of the model in this study. The (Fig 5) below shows a typical characteristic model for the Kafka cluster to analyze the information and produce the analytically proved result. The framework works on the partitioning process, and results are generated based on the topics specified in the cluster. The proposed model only partially depends on Kafka for the analytics; however, due to its robust and secure nature with strong computational algorithms, Kafka is supposedly one of the important tools in analytics [53] Table 2 shows a technical comparison between Apache Kafka and other data analytics technologies.

**Table 2:** Technical comparison between data analytics technologies.

No.	Technology Name	Key Terms
*1*	*Apache Spark*	*Works based on Master/Slave Architecture*
*2*	*Hive*	*Built on top of Hadoop*, *which provides data query and analysis*
*3*	*Apache Kafka*	*Managing real-time data*, *based on the collection of topics and separated into partitions*

The decision for the patient diagnosis and further evaluation is generated using ML algorithms. Finally, the information is sent to the data visualization stage. It is worth mentioning that computing is used in this case, where machine learning and data analytics take place with the help of fog gateway devices [54]. This ensures the security and safety of the data, which is rooted inside the machine learning pipeline stage.

Table 3 below compares the data mining techniques that are commonly used in various data analysis pipelines. Various cloud service providers provide the services for free or for miners to charge for acquiring the data in the proper prescribed format [55]. The model can use either of the services irrespective of the design, depending upon the experience of the designer who will create the data acquisition pipeline.

**Table 3:** Technical comparison between data mining technologies.

No.	Technology Name	Key Terms
*1*	*RapidMiner*	*An integrated environment for the ML*. *DL*
*2*	*ElasticSearch*	*Distributed environment with ElasticSearch Server and based on PostgreSQL*

Development Pipeline: Once the information is completely available in the format prescribed, it passes onto the development pipeline. This is the main section responsible for our proposed model [56]. The data, received from various IOT devices and finally compressed to the format required, is segregated in the form of the data type—for example, images, audio files, visual files, text information, etc. The model, prescribed in our case, handles three different data types as inputs. Image data are augmented and passed to pre-trained models to confirm the diagnosis of the COVID-19 patient. Various X-rays and diagnostic reports in the form of images are passed in this section. The deep learning classification algorithms ensure that the pre-trained datasets are highly responsible for predicting the nature of disease from the existing inputs. The classification finalizes the information as usual, infected, or any other disease based on the nature of the input. The audio datagram is also one of the integral parts of the inputs taken from various devices. These devices record inputs from the patient’s biometric features [57]. The extraction and selection are done based on various machine learning models for the information from the audio device files. This information is sent to the classification node, where further diagnosis or analysis occurs.

Finally, text data can also be a part of the inputs collected by various IOT devices. This includes information such as a patient’s temperature, oxygen level, heart rate, sugar diagnosis, blood pressure, and more [58]. The information received in this case is more textual and is given to the training model with the help of any machine learning algorithm. Finally, testing and training take place, which gives the final classification of the three existing modes. It must be mentioned that the information received after the classification is done in the development pipeline must be visualized for ease of understanding. The visualization of the information received from the classification and development pipeline can be done with the help of various visualization tools. Many cloud service providers provide services for various data analysis tools. This analysis results in beautiful dashboard-based reports that are easy to compile and read. Two well-known are compared in Table 4 below, which can be used for analysis without cloud services. For ease of convenience in our model, cloud-based subscription services or stand-alone services like Tableau or Plotly can handle the data [59].

**Table 4:** Technical comparison between data visualization technologies.

No.	Technology Name	Key Terms
*1*	*Tableau*	*Business intelligence tool used for visualization*
*2*	*Plotly*	*Plotting or graphs the data*

Based on the overall elaboration in Big Data technologies, Data Architectures, and AI techniques, these technologies are helpful in overcoming the pandemic in a very smart way using minimum resources. Large- lume data can be stored using data architectures, and large-volume data can be executed using AI technologies. This approach provides the best solution to overcome the pandemic and facilitate people’s smartness.

### 4.3 Proposed system case study

**Scenario:** The proposed model was needed to design and develop the system, especially for the COVID-19 disease analysis and detection. Based on the current case study scenario, the focus was to address COVID-19, where blockchain and IoT devices collect the data and secure the dataset via blockchain. Many case studies could be related to that domain, such as disease surveillance and reporting, patient monitoring and management, data security, and privacy. Based on the disease diagnosis, the given scenario was needed to provide the solution using the latest technologies, such as Big Data, AI, and IoT devices for data collection. Patient data security and privacy were the primary concerns, as multiple patients lost their data, which was provided in the healthcare system for diagnosis. The latest technologies can provide the best valuable solution under reasonable choices.

**Outcome:** This case study will provide a valuable solution for using data protection and privacy technologies. The proposed solution was based on the latest technologies, which can provide reliable performance and efficiency in the use of technologies.

**Evaluation:** This case study was evaluated to see the performance of healthcare systems where different IoT devices are used to collect patients’ records. It was examined to see whether the patient’s data is secured by using the Blockchain security feature and is trustable when multiple people are already using these services for different purposes. The proposed research study was qualitative, so we mentioned that it performed well.

## 5 Experimental setup and discussion

The proposed model in this study comprises four basic layers of work. For the model’s simplicity, we have established a simple prototype to handle the data acquired from the open-source data repositories for COVID-19—sample information [60]. The datasets were downloaded from the GitHub repository due to the complexity of the IoT devices and information processing unit. The entire dataset was recognized with the help of machine learning algorithms with the help of data analytics tools. The analysis was done with the help of Kafka as prescribed in the schema in (Fig 5) above. The open-source data repository comprises 930 images from 461 patients. This city-enabled chest X-ray dataset was analyzed using the rule base. The image was categorized into parameters: normal, infected, or something extra. Once the decision-making was done. The information was passed for visualization. The visualized data set could tell a comparative study between the infected and the other side.

**Fig 6:**
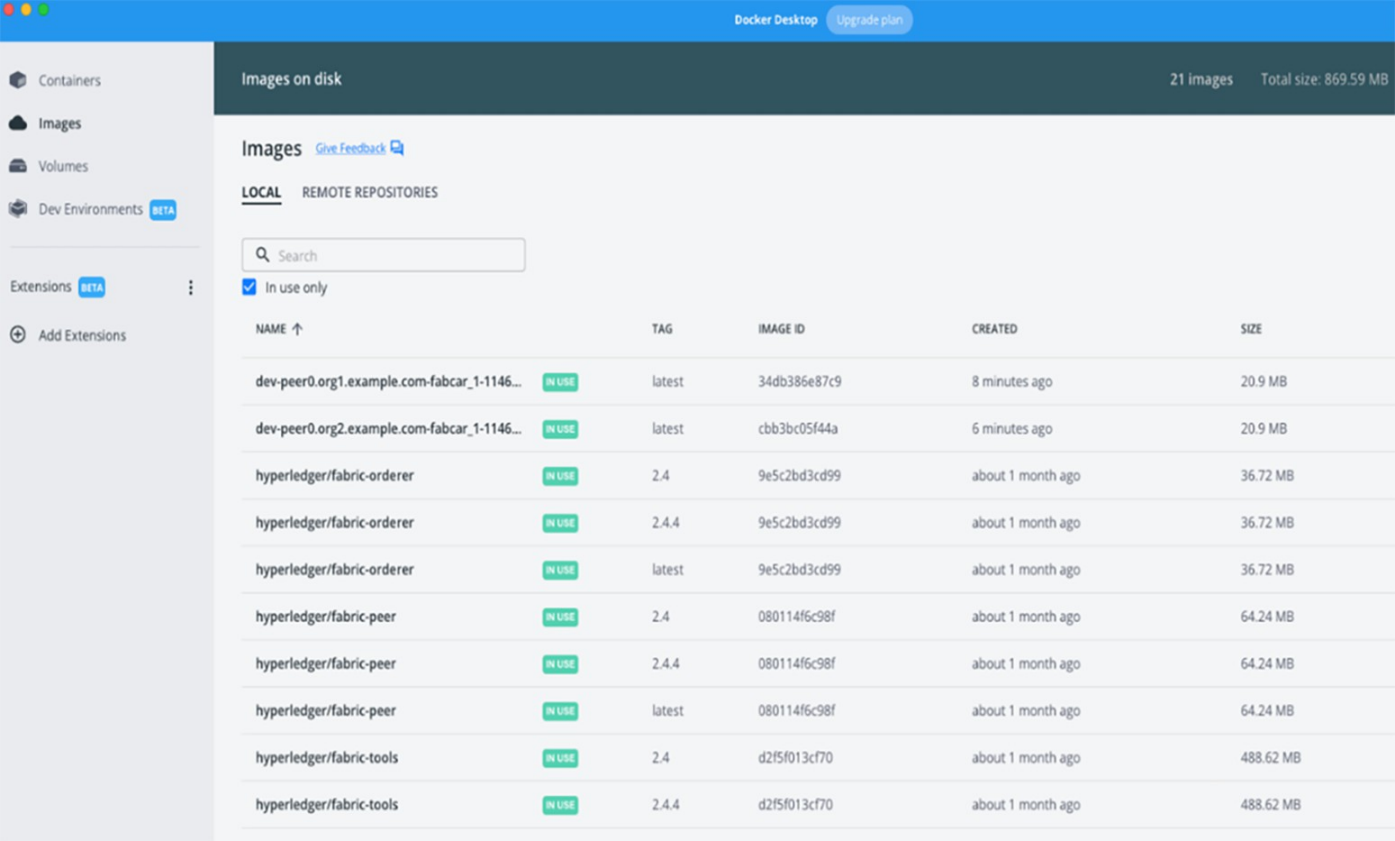
Blockchain nodes for two communicating peers running on hyper ledger fabric.

The core of the dataset model and the data architecture comprises certain features like security and privacy. For the sake of understanding and prototyping the data model, we have deployed a local test network with the help of the University network. as shown in (Fig 6). This test network was committed to a hyper ledger for blockchain. The hyper Ledger product is called fabric. Transaction management will occur based on the exchange of smart contracts once the hyper-ledger fabric is accepted. In the test network after the deployment, we created two channels or organizations that can exchange information as per the model. The two peers who will contact and exchange information in the blockchain network have to agree upon the smart contract that has been created. The smart contract can advise the rules and regulations during the exchange of information and its broadcasting. The complete exchange occurs once the secure key exchange is done for both the peer nodes.

**Fig 7:**
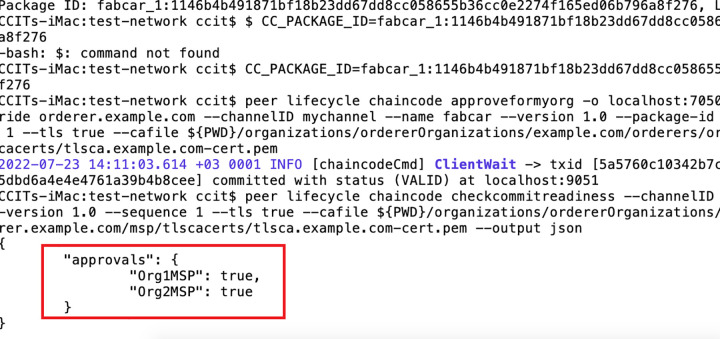
Deployment for the Blockchain Smart Contract at local test network for two organizations.

The (Fig 7) represents the docker images of the two organizations deployed for the smart exchange of data blocks. The information goes up from one peer to another in the test network based on the smart contract exchange. It shows information about the local image running for the two channels responsible for sharing the information across the blockchain. Once the data has been exchanged and smart contracts are received, the data blocks are updated with the information. In the proposed model, the final update can reach the stakeholders based on the blockchain smart contract exchange once a decision is made regarding the information received from the data acquisition layer.

The JSON value for the approval of smart contracts from both peers is shown in the test network (Fig 7) above. Once the information exchange takes place and the smart contracts are verified, the blockchain will update the contents of the block across the decentralized database.

This decentralized information peer node will broadcast the most updated and recent information obtained. This results in security and privacy with the blockchain’s transparency feature. This section discusses COVID-19 analysis and detection approaches: Big Data technologies, Data Architecture and AI techniques, and the blockchain smart contract exchange mechanism.

The proposed model performed a qualitative research study on the latest technologies for COVID-19 data analysis and detection. The deployment of the model is shown in (Fig 7), where the sample test was performed.

### 5.1 Architectural features

Various novel features are depicted after evaluating the proposed model, as discussed below:

- **Binary Class Classification:** The same dataset can classify regular and COVID-19 patients.
- **Multi-Class Classification:** The proposed model diagnoses COVID-19 in two different ways, as it classifies COVID-19 and No-Finding patients. It is also able to distinguish between multi-class Classification (COVID-19 vs. No-Findings vs. another disease).
- **DataOps Utilization:** The proposed architecture is based on DataOps. The DataOps approach is underutilized in the state-of-the-art for managing entire data pipelines for disease discovery.
- **Blockchain Utilization:** According to state-of-the-art studies, no one has integrated blockchain with big data analytics and AI. The proposed model is built on blockchain to decentralize the main dataset repository so every stakeholder can easily access it.

### 5.2 Key values of the proposed model

All stakeholders can easily access the central data repository through the blockchain approach. Transparency in the proposed model will help every stakeholder in decision-making and increase the system’s responsiveness. Furthermore, it allows for the early detection of COVID-19 and supports doctors and healthcare staff in immediately transferring critical condition patients to the right place. The proposed model helps stakeholders share vital information, made possible after employing DataOps with the blockchain approach. As a result, every stakeholder can access the data. The proposed model can overcome the performance issues prevalent with big data. From a software perspective, its capability can be achieved by choosing the right tools and algorithms. The revolution of big data technologies that support parallel computing positively affects performance. The blockchain approach has different lightweight algorithms that can be used to implement the proposed model.

Furthermore, the blockchain approach can significantly qualify the data and accelerate the data analytics and AI processes accordingly. Using high-performance and cloud computing services can also increase the model performance from a hardware perspective. The security attribute can be achieved using the blockchain. The main repository and data sharing are secured to the end user and can only be accessed by a valid user. Thus, there is no doubt that the data is transparent, but every user is managed in the model. One of the most important features of blockchain technology is decentralization, which makes all the data available to all authorized users in an encrypted and secure form.

### 5.3 Mutual benefits of blockchain and big data

People rely on various key points in blockchain technologies and big data. The main reason is that data is rapidly increasing, and traditional intelligence tools cannot perform actions against large volumes of data. Big Data technologies facilitate large volumes, and the emerging field of blockchains is helping data analytics tools. Blockchain and Big data are the best options for large amounts of data. Blockchain facilitates decentralizing the central repository, making the distributed network secure and valuable [31]. High performance and accuracy are assumed without human intervention. No third-party verification costs are required to handle blockchain P2P network decentralized architecture. The transparent nature is quite apparent in preserving security and privacy. Transactions are committed to the ledger with the help of sophisticated mechanisms and digitally signed and encrypted documents. The decentralization makes it robust enough to avoid interference and unethical data acquisition.

Peer-to-peer (P2P) is the key term used in the blockchain decentralized approach, where the communication process works smoothly. File sharing is a feature in P2P, where data collection can be performed quickly and the decentralized platform accessed very quickly. P2P networks are so powerful that they execute enormous volumes of data. Another example is a decentralized platform with valuable encryption, trust mechanisms, and IP configuration features.

### 5.4 Big Data and blockchain opportunities

Blockchain in the Big Data is an emerging combination used in many fields to address complex problems. Blockchain with Big Data technologies helps in various fields where there is a large amount of data that must be valued and secured, either operationally or analytically. Blockchain provides good opportunities for almost every field. It provides opportunities in healthcare, property records, banking, and other fields. Blockchain is a new technology for managing electronic data and potentially supports transparency and accountability [32]. Blockchain provides a ledger property in which all the connected members within the network can see the data. It can be utilized for identity management in healthcare units. Patient consent is required to produce the data online. Blockchain privacy allows patients who do not wish to share their data to keep the information confidential. Data with proper timestamps is available in the blockchain to provide a proper timeline for a series of events.

### 5.5 Challenges of blockchain and big data in deployment

Blockchain is valuable and used on a large scale, but a few points identify limitations where improvements are still needed. We are dealing with large volume, velocity, and variety of data. Using Big Data with blockchain technology may need help with security, response time, and low transaction throughput [9, 61]. The blockchain technology is still very recent. There are still possibilities for unauthorized access or hacking unless the technology matures enough to provide what is needed. International-level regulations and rules still need to be clarified and implemented in the context of blockchain. Since millions of data transactions occur in the blockchain network, The processing capability of the servers needs to be managed and handled. The complex structure sometimes puzzles the end-users when working with the blockchain networks. The stakeholders must receive proper training on how to use such decentralized architectures. The network can sometimes be slow and time-consuming due to low bandwidth or network congestion.

### 5.6 Big data and blockchain benefitial in deployment

Blockchain can be considered a big data analytics technology. Big Data deals with the large volume, variety, and velocity of data. At the same time, there is a need to decentralize the central data repository and create a network that helps every user in the network to access and use the required data. Without blockchain technologies, it will not be easy to analyze the large amount of data created in places like the banking system, healthcare, and property records, as discussed previously. Blockchain ensures that the big data repositories are secure and valuable.

During the model deployment for the large volume dataset, there are some challenges, such that there are still some points where issues arise for the limitation. The solutions could be based on the properties of the blockchain features.

### 5.7 AI and blockchain convergence in healthcare: Opportunities and real-time challenges comparison

Blockchain technology increases security, protects data privacy, and enables operations on healthcare data. AI is used to analyze data and make predictions based on the analysis. Data is central to AI, and AI can collaborate with blockchain. Correspondingly, blockchain increases collaboration and data-sharing security. Blockchain ensures trust in shared data and AI extracts insight and direction from data. Most data collected in healthcare is from public platforms, surveys, or other public data. It is necessary to create some security for a smooth flow of data. AI and blockchain ensure this task is fulfilled in healthcare. Data flowing within healthcare includes information on budget, personnel, legal documents, logistics, medical workflows, and procedures. Internal control and performance should be enhanced to reduce the risk of any mishandling of information. This research signifies the importance of smart information sharing among people through the healthcare system.

Blockchain is a system that records information and stores it, making it impossible to change or hack the system. It is currently essential in digitized systems as it creates a decentralized system that gives every person access to the information simultaneously. It is a distributed database with Bitcoin, a decentralized electronic currency, a well-known application of the concept. We also have AI, entirely computer-based engineering that can relieve additional work stress on humans via automation. The algorithms are used to predict trends and decisions.

An example would be Alzheimer’s disease, for which AI can perform a diagnosis faster than humans. Healthcare is an early adopter of the integration of blockchain and AI to enhance the capabilities of services. Voluminous data is streaming from various sources into the centralized nodes on servers to yield a diagnosis. This data increases with time and converts to big data. Integrating the system to handle such an enormous amount of data is one of the highlighting features of blockchain in healthcare. Biomedical research involving data and AI predictions will be helpful for researchers in finding solutions to various diseases. The model’s training can be done on a vast scale to acquire the disease with persisting symptoms and handle some new symptoms to discover the amendments in the ailment of the person. The research on Covid 19 is well spread and going across the world. This research needs to find a probable system capable enough to predict the nature of disease when a person suffers from the symptoms of corona. The fast and complete census of the patient data is available at the fingertips with the help of proper blockchain integration. Fast-tracking for diseases like diabetes and blood pressure can be done very quickly with the help of advanced deep-learning algorithms and machine-learning procedures.

There are various opportunities related to this blockchain and big data integration. However, various challenges can be faced regarding the above context. Hacking or malicious software can cause security flaws that may cause identity and data theft. Many incidents of this vicious attack have happened in developed countries. Privacy and integrity of data are paramount. Patient data privacy can be compromised due to the large flow of information required for fast services. Blockchain handles the data so that it becomes impossible to identify the specific data. Many computers and blockchain devices are required for implementation in the blockchain system. This may increase the system’s budget. This challenge concerns the system’s size, which is necessary for holding and sharing patients’ information. Blockchain technology is a new technology, the latest technology, and there are many areas for improvement. There are still some problems with transaction confirmation and real-time responses, and it takes time to confirm a transaction. At present, we are mainly using AI in X-rays and CT scans. So integrating it with blockchain may take work. It may result in some unpredictable results. The main challenge relates to expenses, which may cause hospitals additional costs. Making use of information is very important. Interoperability refers to the ability of different computer systems to connect and exchange information. This may seem easy, but it is very challenging. Scientists are working on easing the exchange of information between blockchain and AI, but more is needed.

### 5.8 Ethical implecation of using AI and blockchain in healthcare

Developing a passion for using AI and blockchain technologies in healthcare is ethically necessary. Without any justification or authorization, no one can deploy AI technologies in healthcare. So after the successful completion of the proposed research study, it has to sign the agreement for mutual cooperation for using such a model in the healthcare domain.

Advancements in technology have had a significant impact on every field of life. To ensure safety, security, and data protection, an agreement should be needed. Healthcare is the most crucial domain where maximum public dealings occur, and there should be security and deployment of the model in real-time.

## 6 Conclusion

The study presented in this article formulated a model that can manage the COVID-19 patient data and predict the nature of ailment using artificial intelligence. The entire model was divided into four layered architectures, each comprising specific functionality. The basic data acquisition structure was done using various IoT-enabled devices or medical notes from healthcare workers. Data storage in various formats is executed in the second stage with the help of data storage architectures. For the prescribed model, the proposed research study used the datasets available as open source and analyzed them using data analysis techniques. The third layer empowers the model to process the data acquired and store it in the previous layers. Based on solid machine learning algorithms and deep learning paradigms, the model decided to classify the patient’s ailment. The final stage comprised the decentralized model approach in the P2P network. The blockchain for health statistics could broadcast the data analyzed and decisions made for the patient. The study works with a prototype model presented at the local test network where the two peers exchange smart contracts. After the exchange of the smart contract, the final block of information is pushed into the ledger. The entire model works in data acquisition, storage, decision-making, and, finally, pushing it to the blockchain node. The resulting model provides good results for identification of data and COVID-19 information.

The decentralized nodes allow storing and handling bulk amounts of data received from various edge gateway devices. Finally, the security and privacy of the user information is managed within the blockchain. The overall model is suitable for managing big data from various IoT-based devices and deciding the nature of an ailment using artificial intelligence and machine learning. The proposed research study could make more varied use of the technologies to facilitate people all over the globe using IoT devices. AI-integrated solutions with the IoT and blockchain could be provided, and the proposed feasibility study will approve the system’s deployment in the real world. Based on the proposed research study, a comprehensive review of their data architectures, IoT device usage, and the blockchain’s security helps implement the same model in real-world scenarios. That model can help to provide the best understanding of COVID-19-related diseases.
